# Imaging at the nexus: how state of the art imaging techniques can enhance our understanding of cancer and fibrosis

**DOI:** 10.1186/s12967-024-05379-1

**Published:** 2024-06-13

**Authors:** Alireza Baniasadi, Jeeban P. Das, Conor M. Prendergast, Zahra Beizavi, Hong Y. Ma, Muhammad Yaman Jaber, Kathleen M. Capaccione

**Affiliations:** 1 Department of Radiology, Columbia University Irving Medical Center, 622 W 168Th Street, New York, NY 10032 USA; 2 Department of Radiology, Memorial Sloan Kettering Cancer Center, New York, NY 10065 USA; 3 Department of Radiology, Damascus University, Damascus, Syria

**Keywords:** Cancer, Fibrosis, Imaging techniques, Diagnosis, Tumor microenvironment

## Abstract

Both cancer and fibrosis are diseases involving dysregulation of cell signaling pathways resulting in an altered cellular microenvironment which ultimately leads to progression of the condition. The two disease entities share common molecular pathophysiology and recent research has illuminated the how each promotes the other. Multiple imaging techniques have been developed to aid in the early and accurate diagnosis of each disease, and given the commonalities between the pathophysiology of the conditions, advances in imaging one disease have opened new avenues to study the other. Here, we detail the most up-to-date advances in imaging techniques for each disease and how they have crossed over to improve detection and monitoring of the other. We explore techniques in positron emission tomography (PET), magnetic resonance imaging (MRI), second generation harmonic Imaging (SGHI), ultrasound (US), radiomics, and artificial intelligence (AI). A new diagnostic imaging tool in PET/computed tomography (CT) is the use of radiolabeled fibroblast activation protein inhibitor (FAPI). SGHI uses high-frequency sound waves to penetrate deeper into the tissue, providing a more detailed view of the tumor microenvironment. Artificial intelligence with the aid of advanced deep learning (DL) algorithms has been highly effective in training computer systems to diagnose and classify neoplastic lesions in multiple organs. Ultimately, advancing imaging techniques in cancer and fibrosis can lead to significantly more timely and accurate diagnoses of both diseases resulting in better patient outcomes.

## Introduction

**Fig. 1:**
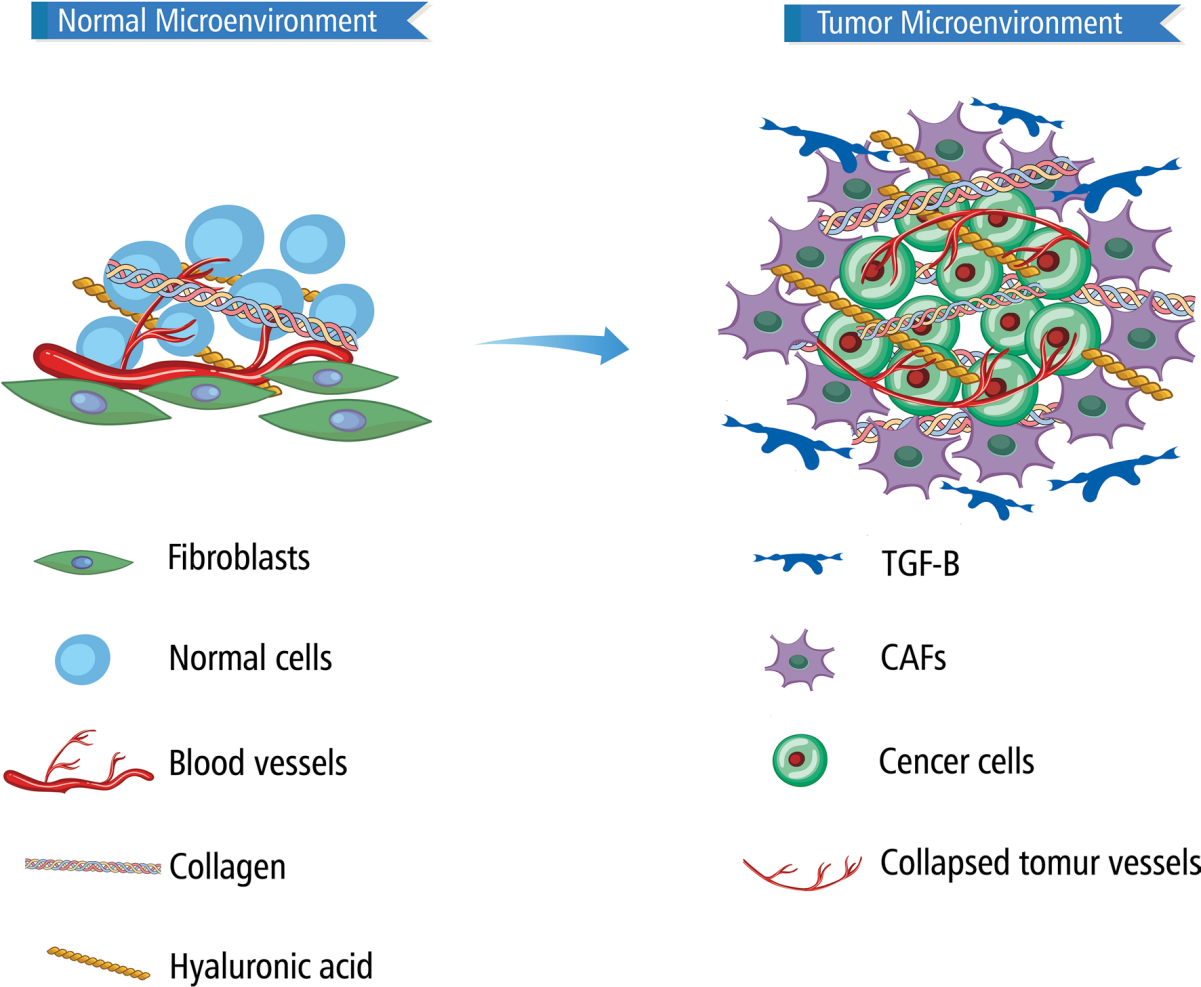
Normal cells are surrounded by blood vessels, collagen fibers, fibroblasts, and other extracellular matrix components. However, during tumor development, CAFs and TGF-β cause ECM alteration, leading to the formation of a stiff fibrotic layer around tumoral cells. This microenvironment facilitates the growth, invasiveness, and treatment resistance of tumoral cells

Uncontrolled inflammation plays a considerable role in numerous diseases, including fibrosis and cancer [1]. In addition, fibrosis and cancer have common mechanisms, risk factors, and cellular connections. The presence of fibrosis in specific organs can be a precursor to developing corresponding malignancies, such as hepatocellular, lung, gastric, head and neck, colon, pancreatic, cervical, and vulvar cancers [2–4]. Conversely, cancer can cause the growth of dense fibrous tissue, known as desmoplasia, a critical pathologic feature of tissue injury, most notably in the pancreas, liver, and lungs [1, 5]. As such, the pathophysiology of both diseases has significant overlap, which manifests at both micro and macro scopic levels. Major tumor microenvironment (TME) components including stromal cells, immune cells, cancer-associated fibroblasts (CAFs), and noncellular components of the extracellular matrix (ECM) such as collagen, hyaluronan, and fibronectin, significantly contribute to fibrosis, promoting tumor progression, metastasis, and resistance to therapy [6–8]. These cells and cellular mediators also play a role in fibrotic diseases of multiple organs. CAFs exhibit significant heterogeneity depending on the stage of cancer, which may affect treatment outcomes. The prevalence of CAF subtypes in a tumor or metastatic site varies in response to different states, including inflammatory, precancerous, and malignant states, as well as therapy [9, 10]. Additionally, they are the main source of transforming growth factor β (TGF-β) overproduction in cancer [11]. TGF-β is a critical pro-fibrotic cytokine, and expression results in fibrosis, or scarring down of the tumor area in various organs such as the liver, lung, kidney, breast, and others [12, 13]. The effects of TGF-β are cell type in context dependent. When normal conditions exist, TGF-β promotes homeostasis, maintaining epithelial integrity, and anti-tumor effects such as inhibition of cell proliferation, inhibition of inflammation, and induction of apoptosis, however in a disease state it stimulates the activation of fibroblasts and subsequently triggers inflammation, angiogenesis, fibrosis, and basement membrane invasion [14]. Through inducing the expression of collagen which is deposited into the microenvironment and reducing the expression of extracellular matrix proteases, TGF-β causes the maintenance and continuation of the cycle of progressive fibrosis when cellular conditions are promoting a pro-fibrotic state [5] Fig. 1.

Computed tomography (CT) scan is a simple and cost-effective means to diagnose many diseases. CT scanners generate cross-sectional images of the internal structures by rotating an X-ray source and detector around the body and combining advanced computing for image processing. The early CT scans, which were a revolution in medical imaging, had limited image quality, longer scanning time, and higher doses of radiation [15]. Improvements in nearly all aspects of computer tomography have allowed for widespread access to high-quality cross-sectional imaging. The Hounsfield unit (HU) is a measurement of radiodensity used in CT imaging. This unit ranges from − 1000 HU for air to 3000 HU for dense bone, with distilled water (at standard pressure and temperature) considered zero Hounsfield units. Dense tissue appears brighter because it absorbs more X-rays, and less dense tissue appears darker because it absorbs fewer X-rays. Fibrosis appears as an area with increased density in the target organ due to the high deposition of collagen and extracellular matrix components resulting in more radiographic density. Thus, it has a higher Hounsfield unit compared to normal tissue [16].

With advances in image acquisition and analysis, various techniques have been developed to improve accuracy and decrease acquisition time for CT. One of the limitations of CT is soft tissue discrimination, which can limit the evaluation of fibrotic tissue from normal regions. To overcome this limitation, contrast can be used to enhance soft tissue visibility on CT. In fibrotic diseases, collagen deposition alters tissue architecture, leading to changes in blood flow patterns and vascular remodeling. The contrast agents, which are usually based on iodine, reach the blood vessels of the target area and increase resolution between the blood vessels and the surrounding tissues [17]. Additionally, post-processing techniques can also be used to improve diagnostic capabilities of traditional CT and reduce complications such as contrast reactions and excess radiation exposure. One of these post-processing techniques is deep learning (DL), which is a sub-branch of artificial intelligence (AI) that can automatically analyze a large amount of data and extract features, discussed further in the dedicated section [18].

In this review, we explore new ways in which diagnostic imaging can enhance our ability to visualize and diagnose fibrosis, and how the same modalities can improve our diagnosis of cancer. Commonalities at the molecular level between these diseases will allow us to employ technologies developed for one to better diagnose and follow the other.

## PET imaging

Molecular imaging with positron emission tomography (PET) enables in vivo visualization of functional processes within a tissue or organ of interest using targeted molecular probes and can add significant value to conventional imaging in both the assessment of cancer and fibrosis. It is being increasingly used to assess pulmonary fibrosis (PF) in select patients.

### Applications for *cancer* imaging

CT plays an important role in the initial staging of lung cancer, however, molecular imaging with positron emission tomography (PET)/CT may detect and characterize additional lesions, with the potential to provide critical prognostic information and alter management [19].

The most ubiquitous radiotracer in PET/CT lung cancer imaging is 18-fluorine (^18^F) fluorodeoxyglucose (FDG), with several metabolic parameters used to quantify tumor aggressiveness and assess prognosis, including maximum standardized uptake value (SUV_max_), metabolic tumor volume (MTV) and total lesion glycolysis (TLG). Higher values of these parameters are predictive of a decreased survival in patients with surgical lung cancer and advanced lung cancer and may be used for risk stratification in disease control and survival. Patients with tumors that exhibit intense FDG uptake may be considered at a high risk of treatment failure and may benefit from more aggressive treatment [20–23]. Although FDG is the most established radiotracer for evaluating lung cancer, it has several limitations, including poor specificity [24].

For this reason, several novel radionuclides have been developed for the evaluation of lung cancer patients including ^18^F-fluorothymidine (^18^F-FLT), ^18^F-fluoromisonidazole (^18^F-FMISO), targeted integrin imaging and most recently, radiolabeled fibroblast activation protein inhibitors (FAPI) [25].

FAPI molecules have recently been explored as potential targets in molecular imaging for several cancers. Fibroblast activation protein (FAP) is a stroma-specific marker and is overexpressed by activated fibroblasts including cancer associated fibroblasts (CAFs) and fibroblasts occurring in other disease states. CAFs are cells found in the tumor stroma and play a crucial role in tumor growth and aggressiveness [26]. Therefore, radiolabeled FAPI can be utilized as a novel imaging tool to visualize fibrosis and the tumor microenvironment architecture. In a head-to-head performance comparison of ^68^Ga-FAPI PET/CT and ^18^F-FDG PET/CT in patients with lung cancer on a lesion-by-lesion basis, authors found that ^68^Ga-FAPI PET/CT demonstrated a better staging performance in lung cancer patients with different pathological stages, especially those with localized disease [27]. In addition, ^68^Ga-FAPI has been shown to identify more suspected nodal, pleural, osseous, and intracranial metastases than ^18^F-FDG in patients with lung cancer imaged with PET/CT and both radiotracers [28]. In another study conducted by Chen et al., ^68^Ga-FAPI PET/CT showed better sensitivity and accuracy in detecting various types of primary tumors, such as liver and nasopharyngeal tumors, etc., as well as in metastatic lesions including bone and visceral metastases, and metastatic lymph nodes, compared to ^18^F-FDG PET/CT [29]. Also, Kömek et al. demonstrated that ^68^Ga-FAPI PET/CT had higher SUV_max_ values to detect primary breast lesions compared to ^18^F-FDG PET/CT. They reported a sensitivity of 100% and specificity of 95.6% for ^68^Ga-FAPI PET/CT, while ^18^F-FDG PET/CT had a sensitivity of 78.2% and specificity of 100% [30].

Prostate-specific membrane antigen (PSMA) PET is another powerful emerging tool used for primary staging, recurrence, and advanced disease in prostate cancer. ^68^Ga-PSMA-11 and ^18^F-PSMA-1007 PET/CT are widely used in these clinical settings. In head-to-head analyses, ^18^F-PSMA-1007 showed higher sensitivity (100% vs 85.7%), accuracy (94.5% vs 93.3%), and lesion SUV_max_ but lower specificity (90.9% vs 98.2%) compared to ^68^Ga-PSMA-11 for detecting dominant lesions. The positive predictive value was also lower for ^18^F-PSMA-1007 (87.5% vs 96.8%), but it had a higher negative predictive value (100% vs 91.5%). Both tracers were effective in detecting the dominant lesions, but ^18^F-PSMA-1007 showed superior performance in identifying focal lesions compared to ^68^Ga-PSMA-11 [31, 32]. Also, another radiotracer, ^64^Cu-DOTA-AE105, is designed to target the human urokinase-type plasminogen activator receptor (uPAR) expressed in cancer cells. It is used to improve prostate cancer diagnosis, diagnose aggressive cancers, and determine cancer aggressiveness [33–35].

In a recent phase 3 clinical trial, the effectiveness of ^89^Zr-DFO-girentuximab PET/CT was assessed in 288 patients with clinical stage T1 (< 7 cm) solid renal masses to differentiate ccRCC from other kidney lesions. Participants in the study received ^89^Zr-DFO-girentuximab PET/CT and then underwent partial or radical nephrectomy for pathology. The majority of patients had ccRCC (67%), followed by papillary RCC (15%), chromophobe RCC (8%), and the remaining had benign and malignant tumors. The sensitivity, specificity, positive predictive value (PPV), negative predictive value (NPV), and accuracy of ^89^Zr-DFO-girentuximab PET/CT were reported as 85.5%, 87%, 93%, 75%, and 86%, respectively, indicating promising results [36].

Numerous other PET/CT radiotracers have been used in oncology, and our detailed in Table 1. The radioisotopes that are used for PET imaging are also shown in this table. The shorter-lived isotopes such as ^11^C, ^68^Ga, and ^18^F are ideal for labeling peptides and other small molecules, that quickly clear from circulation within minutes to hours. As well as, ^18^F has low positron energy compared with the other nuclides, which provides the highest resolution of images obtained [37, 38]. Isotopes such as 64Cu and 76Br have intermediate half-lives, which 64Cu is suitable for many types of molecules [39]. On the other hand, longer-lived isotopes like ^89^Zr and ^124^I are well-suited for labeling antibodies, their fragments, and nanoparticles, that remain in circulation for hours to days to reach their targets. Additionally, ^89^Zr doesn't require highly enriched targets, has lower production energy, and doesn't cause radioactive uptake in non-targeted organs compared with ^124^I [40].

**Table 1:** PET/CT radiotracers in oncology:

Radionuclide	Decay	Radiotracer	Application	FDA approved
Fluorine-18	T_1/2_ = 109.8 m β^+^ = 96.7%	^18^F-FDG	Used for diagnosis, staging, and management of various types of cancer	*
		^18^F-FLT	To diagnose, stage, and assess response to therapy; differentiate tumors from inflammation, and report on cell proliferation [198–200]	*
		^18^F-FMISO	Tumor prognosis, predict metastasis, and evaluate hypoxia in tumors [201, 202]	*
		^18^F-FSPG	Diagnosis of primary intracranial tumors and malignancies; measures X_c_^−^ transporter activity which is overexpressed in different types of tumors [203–206]	–
		^18^F-FBEM	Used for insulinoma imaging; detection of malignant lesions with high EGFR activity, monitoring of metabolic activity and leukocyte recruitment [53, 207, 208]	–
		^18^F-αvβ6-BP	Diagnosis of primary and metastatic lesions including lung, liver, and brain; targeting integrin αvβ6, which is overexpressed in cancer and fibrosis [209, 210]	–
		^18^F-PSMA-1007	Used for diagnosing, monitoring recurrences, and detecting metastases particularly nodal metastases in prostate cancer; prostate-specific-membrane-antigen (PSMA)-based radiopharmaceutical [31, 32, 211–213]	*
		^18^F-alfatide	Used for detecting breast cancer, predicting the outcome of CCRT in advanced NSCLC, and assessing liver fibrosis progression; a tracer which binds to αvβ3 [66, 214, 215]	–
		^18^F-fluciclovine	Diagnosing prostate cancer, breast cancer, liver metastases, brain tumor; increased in tumor cells by amino acid transporters [216–219]	*
Gallium-68	T_1/2_ = 67.8 m β^+^ = 88.9%	^68^Ga-PSMA-11	Diagnosis and staging of prostate cancer and occult biochemical recurrence [31, 220, 221]	*
		^68^Ga-FAPI	Diagnosing pancreatic cancer, for detecting primary gastric cancer, post-treatment recurrence and metastasis, lung cancer and fibrosis [27, 222, 223]	*
		^68^Ga-ABY-025	For diagnosis of breast cancer and metastasis; evaluation of HER2 expression (224, 225)	–
Iodine-124	T_1/2_ = 4.2 d β^+^ = 23%	^124^I-girentuximab	Detection of Renal Cell Carcinoma [226]	–
Bromine-76	T_1/2_ = 16.2 h β^+^ = 57%	2-^76^Br-BAMP	Detection of various tumors such as lung and brain tumors, lymphomas, and melanomas [227]	–
Zirconium 89	T_1/2_ = 3.27 d β^+^ = 23%	^89^Zr-bevacizumab	Recurrent glioblastoma, Breast cancer diagnosis and lymph node metastasis; VEGF-A overexpression [228, 229]	*
		^89^Zr-DFO-girentuximab	Renal cell carcinoma diagnosis, differentiation between ccRCC and non-ccRCC lesions; Carbonic anhydrase IX antigen which is overexpressed in ccRCC [36, 230]	–
		^89^Zr-rituximab	B Cell Lymphoma; targeting CD20 [231]	–
Carbon-11	T_1/2_ = 20.4 m β^+^ = 99.8%	^11^C-acetate	Bladder cancer, Prostate cancer diagnosis, recurrence prognosis, and detection of metastasis; hepatocellular carcinoma diagnosis; an indicator used to track cytoplasmic lipid synthesis which increased in tumors [232–236]	–
		^11^C-choline	Detection of prostate cancer and recurrence [237, 238]	*
Copper-64	T_1/2_ = 12.7 h β^+^ = 17.5% β^−^ = 39%	^64^Cu-DOTA-AE105	Prostate cancer diagnosis, cancer invasion prognosis, a tracer for urokinase-type plasminogen activator receptor expression level (uPAR) [33–35]	–
		^64^Cu-PSMA	Prostate cancer diagnosis [239]	–

### Applications for fibrosis imaging

While the utility of PET is well-established in lung cancer imaging, its role in clinical diagnosis of PF is less well defined. Despite the lack of integration into the standard treatment algorithm, fibrotic changes of the lungs are well visualized on FDG and may be seen prior to CT changes [41, 42]. Conventional imaging with high resolution (HR) CT can demonstrate advanced disease, as characterized by lung honeycombing, reticulation and architectural distortion [43, 44], but early disease stages remain difficult to identify, especially in the context of isolating those who may develop more progressive and rapidly fatal forms of PF. Molecular imaging can convey additional benefits as a potential non-invasive early biomarker of PF, as obtaining lung tissue carries considerable risk for patients, often precluding its use for investigational purposes [45].

Several conventional radiotracers as well as novel radiolabeled probes that have been evaluated for the purpose of assessing PF. The most ubiquitous radiotracer, ^18^F-FDG, has shown moderate success in assessing fibrotic lung disease, however with limited specificity as uptake cannot distinguish between inflammation, fibrosis, and malignant cell proliferation [41, 46–51].

In one of the earlier studies evaluating the utility of ^18^F-FDG PET/CT in PF, Groves et al. assessed 36 consecutive patients and calculated the SUV_max_ to assess maximal pulmonary FDG metabolism and correlated uptake to HRCT lung findings. Increased FDG metabolism was seen in all patients and pulmonary FDG uptake predicted the measurements of health and lung physiology. Of note, FDG avidity was higher when the site of maximal uptake corresponded to areas of reticulation or honeycombing on HRCT, compared to those with ground-glass patterns [46].

Win et al. assessed the reproducibility of ^18^F-FDG PET/CT in patients with PF and demonstrated excellent short-term reproducibility as well as excellent intra-observer agreement with some interobserver bias, suggesting that a single observer would facilitate optimal imaging follow-up [47]. Win also investigated the potential of ^18^F-FDG-PET/CT to predict mortality in PF, evaluating 113 patients using several PET parameters including SUV_max_, background lung activity (SUV_min_), and target-to-background (SUV_max_/SUV_min_) ratio (TBR). During a mean follow-up of almost two and a half years, the authors found that a high pulmonary TBR was independently associated with increased risk of mortality [47].

Justet et al. also evaluated the prognostic impact of ^18^F-FDG PET/CT in PF by assessing both metabolic lung volume (MLV) and TLG in 27 patients. Increased MLV and TLG were independent predictors of death or disease progression during the 12-month period post scan completion, on both univariate and multivariate analysis, suggesting that FDG lung uptake could predict progression-free survival for patients with PF [41]. Jacquelin et al. aimed to evaluate the ability of ^18^F-FDG PET/CT to predict therapeutic response in a cohort of 18 PF patients using SUV_max_, FDG uptake extent as a percentage of lung volume and HRCT fibrosis scores. Extent of FDG uptake was associated with improved pulmonary function under treatment, whereas SUV_max_ and HRCT fibrosis scores were not, with the authors concluding that the quantification of FDG uptake extent might be useful to predict functional improvement in the post-treatment setting [49].

Nobashi et al. investigated the relationships between ^18^F-FDG PET/CT parameters and clinical indicators in PF including the interstitial lung disease (ILD)- sex-age-physiology (GAP) index, by comparing SUV_mean_, SUV_TF_ (defined as corrected SUV_mean_ by using tissue fraction (TF) and mean computed tomography density on PET/CT), and CT_mean_ in 90 ILD patients versus 15 controls. The authors found that PET parameters were significantly higher in ILD patients than in healthy controls and that a higher SUV_mean_ indicated a poorer prognosis, especially in patients with moderate risk based on ILD-GAP index, providing independent prognostic information in patients with PF [50]. Subsequently, Bondue et al. determined whether quantitative assessment of FDG uptake in the lung post initiation of anti-fibrotic treatments pirfenidone or nintedanib could be used as a biomarker to evaluate prognostic significance in a murine model of pulmonary fibrosis. In PF patients, no significant decrease in FDG lung uptake before and 3 months after treatment or at one year of follow up was observed, leading the authors to conclude that there was no utility in clinical practice to assess an early response of PF patients to treatment [51].

More recently, Fraioli et al. investigated the combined performance of quantitative CT (qCT) following a computer algorithm analysis to assess survival in 113 PF patients who consecutively underwent ^18^F-FDG PET/CT imaging and HRCT imaging at a single institution with the authors concluding that both ^18^F-FDG PET and qCT were independent and synergistic indicators in predicting mortality [52]. Bondue et al. also investigated the contribution of inflammation relative to fibrosis by evaluating the pulmonary uptake of FDG on PET/CT in bleomycin-induced PF in murine models. To assess the contribution of inflammatory uptake, the authors comparatively evaluated the signal contribution as a result of leukocyte recruitment in the lung parenchyma using concomitant ^18^F-4-fluorobenzamido-N-ethylamino-maleimide (^18^F-FBEM)-labeled leukocytes. The relationship between different doses of bleomycin, changes in lung collagen content, and level of ^18^F-FDG uptake were analyzed, with the authors noting that lung mean standardized uptake values correlated with bleomycin doses, histologic score of fibrosis, lung hydroxyproline content, and weight loss. The authors concluded that both ^18^F-FDG- and ^18^F-FBEM-labeled leukocyte PET/CT enabled monitoring of metabolic activity and leukocyte recruitment in a mouse model of PF [53, 54].

Another target in both cancer and fibrosis is the chemokine receptor 2 (CCR2). Monocyte and interstitial macrophages that express CCR2 are active in pulmonary fibrosis and can be non-invasively tracked with PET using ^64^Cu-DOTA-ECL1i, as has been shown in mice with bleomycin- or radiation-induced PF as well as in human subjects with PF. Mouse models established that increased ^64^Cu-DOTA-ECL1i PET uptake in the lung correlated with CCR2 + cell infiltration associated with fibrosis and in therapeutic models, while medication related inhibition of fibrosis reduced CCR2 + macrophage accumulation and uptake of the radiotracer in mouse lungs. Human imaging revealed a relative paucity of pulmonary uptake in healthy volunteers, whereas patients with PF demonstrated radiotracer uptake in areas of fibrosis. Brody et al. concluded that these findings supported the potential role for imaging CCR2 + cells in PF to potentially provide a molecular target for both therapy and treatment-response monitoring [55].

Increased deposition of ECM fibers such as collagen, fibronectin, and fibrinogen occur at the onset and during the progression of pulmonary fibrosis and so the targeting of these ECM components using novel radiotracers represent a unique opportunity to identify early disease development. Platelet glycoprotein VI (GPVI) fusion protein plays a critical part in platelet aggregation during wound repair due to its high affinity after dimerization for ECM fibers [56]. GPVI-Fc, an antibody complex protein with an affinity for GPVI dimers can be imaged following radiolabeling with ^64^Cu-NOTA (^64^Cu-GPVI-Fc) [57]. Isser et al. used this ^64^Cu-GPVI-Fc radiotracer targeting ECM fibers on PET to observe longitudinal remodeling in a bleomycin-induced PF mouse model noninvasively to study the potential of the approach in comparison to ^18^F-FDG PET imaging of PF. Of note, ^64^Cu-GPVI-Fc showed significant uptake in fibrotic lungs, matching histology results and in comparison, to ^18^F-FDG PET uptake, ^64^Cu-GPVI-Fc avidity was associated with tissue fibrosis only, and not inflammation [58].

Activated fibroblasts play a pivotal role in the pathogenesis of pulmonary fibrosis by contributing to fibrosis and inflammation following expression of FAP, which is selectively expressed on activated stromal fibroblasts during tissue remodeling and is associated with PF [59, 60]. In addition to the elevated uptake of FAPI tracers in various malignant entities, it can also occur in benign processes, including fibrotic lesions [61, 62].

Rosenkrans et al. determined the utility of FAPI for PET imaging in a mouse model of PF. Following induction of PF via administration of bleomycin, ^68^Ga-FAPI-46 PET/CT imaging was at 7 days and 14 days following disease induction. ^68^Ga-FAPI-46 uptake quantification was recorded, as well as lung CT density in Hounsfield units and histologic examination of PF. While CT only detected differences in the fibrotic response at 14 days post-bleomycin administration, ^68^Ga-FAPI-46 pulmonary uptake was significantly higher in the bleomycin group than in control subjects at both 7 days and 14 days. These findings were consistent with an increase in both fibrinogenesis and FAP expression as seen in histology. The authors concluded that the ability of FAPI PET to detect both the presence and activity of lung fibrogenesis, made it a promising tool for assessing early disease activity in lung fibrosis patients [63].

In other studies, Pirasteh et al. found a strong correlation between ^68^Ga-FAPI-46 uptake in the liver across F2 and F3/F4 fibrosis stages in a preclinical swine model. However, they did not observe a significant difference in the uptake of the baseline liver and the liver classified as F0/F1 [64]. Also, in a preclinical study, Varasteh et al. induced myocardial infarction by coronary ligation in a murine model. They used ^68^Ga-FAPI-04 PET imaging to demonstrate fibroblast activation and observed significant radiotracer accumulation in the infarct zone, particularly in the border of the ischemic area. The maximum accumulation occurred 6 days after MI and gradually receded to baseline by 2 weeks. Subsequently, autoradiography and hematoxylin–eosin staining confirmed that the PET signals correlated with FAP-positive myofibroblasts in the infarct border zones [65].

In another study conducted by Shao et al. focused on investigating the expression and function of integrin αvβ3 on activated hepatic stellate cells (HSCs) in the injured liver. Integrin αvβ3, known as a vitronectin receptor, is responsible for triggering the fibrogenic activation of HSCs. The expression of integrin αvβ3 protein increases as fibrosis progresses in human tissue, and it is predominantly located on activated HSCs. The study demonstrated that ^18^F-alfatide PET imaging exhibits high affinity and specificity towards integrin αvβ3 in both animal liver fibrotic models and human fibrotic liver tissue [66].

### Cross-application of PET imaging between *cancer* and fibrosis

Based on Rosenkrans et al. pre-clinical models [63], it was hypothesized that ^68^Ga-FAPI PET/CT may be a useful imaging and diagnostic tool for PF in humans, not just to assess cancer. Röhrich et al. aimed to evaluate the imaging properties of ^68^Ga-FAPI PET/CT in PF and to confirm FAP expression in fibrotic lesions via biopsy and immunohistochemistry of human samples and in lung sections of genetically engineered mice with an idiopathic pulmonary fibrosis (IPF)-like lung disease. Röhrich et al. evaluated 15 patients with pulmonary fibrosis and suspected lung cancer using ^68^Ga-FAPI-46 PET/CT. The authors recorded the SUV_max_ and SUV_mean_ of fibrotic lesions and lung neoplasms in addition to CT-density and TBR. PET imaging was correlated with CT-based fibrosis scores. Fibrotic lesions as well as pulmonary neoplasms showed markedly elevated ^68^Ga-FAPI uptake and high TBR. ^68^Ga-FAPI uptake showed a positive correlation with the CT-based fibrosis index. The authors concluded that ^68^Ga-FAPI PET/CT imaging is a promising new imaging modality for both PF and lung cancers [67]. In another study conducted by Bergmann et al., 21 patients with systemic sclerosis-associated ILD underwent ^68^Ga-FAPI PET/CT, and the results revealed that FAP imaging can indicate fibrotic activity. The study found that the intensity of FAPi-uptake is associated with pulmonary disease progression, regardless of the extent of involvement in CT scans and lung function at the beginning. Additionally, they observed a reduction in ^68^Ga-FAPI uptake after antifibrotic treatment [68].

Both ^18^F-FDG and ^68^Ga-FAPI-46 have been used for the assessment of cancer and PF in isolation, with further instances where their use has shown the potential to impact management in patients with PF and co-existing lung cancer, especially in the context of acute exacerbations (AE) of PF following treatment. For example, Fukunaga et al. investigated whether ^18^F-FDG accumulation in normal or less-affected lungs with PF increased in 36 lung cancer patients with postoperative AE of PF, compared to 50 patients without PF on pre-operative PET/CT evaluation. ^18^F-FDG-PET/CT demonstrated increased SUV_mean_ as well as elevated SUV_TF_ in normal or less-affected lungs for lung cancer patients with AE potentially reflecting regional fibrosis and inflammatory change [69]. Yamamichi et al. aimed to investigate whether the SUV_max_ was useful in assessing the postoperative risk of AE and severe respiratory adverse events in patients with lung cancer after surgical resection, including a subset of 120 patients with PF. SUV_max_ of the main tumor and that of the non-malignant lung areas were independently associated with both AE and severe respiratory adverse events on multivariable analysis, in both all patients and in the 120 patients with PF [70].

**Fig. 2:**
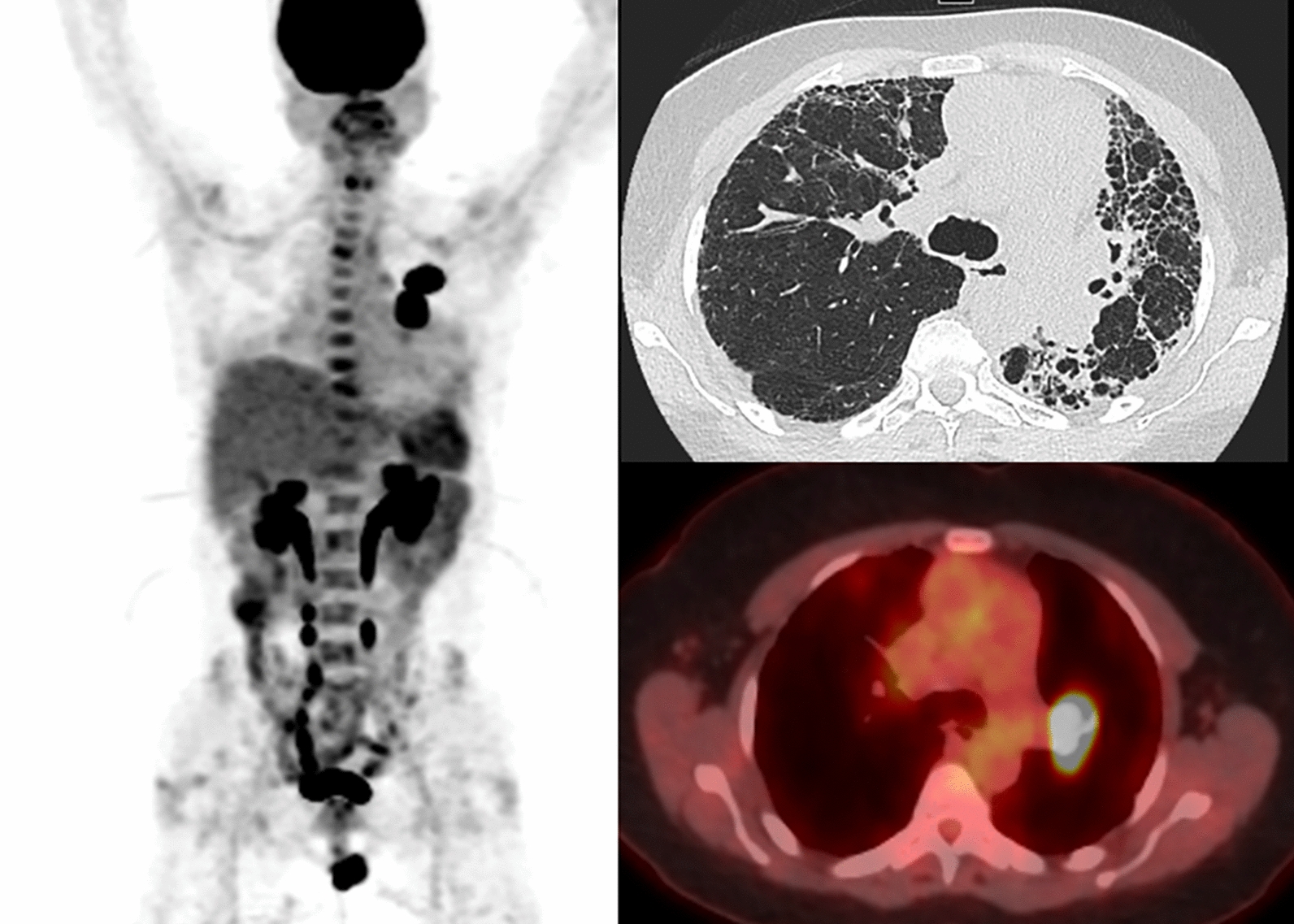
69-year-old man with worsening dyspnea. Maximum intensity projection (MIP), axial CT image and fused axial FDG PET/CT demonstrating heterogeneous FDG uptake corresponding to reticular and linear opacities and areas of honeycombing corresponding to pulmonary fibrosis with focal intense left perihilar FDG uptake corresponding to mass on CT, subsequently biopsied and consistent with adenocarcinoma. Multiple additional FDG avid vertebral lesions were consistent with metastases and CT occult on prior conventional imaging. Low level FDG uptake in the periphery correlates with areas of fibrosis on CT

**Fig. 3:**
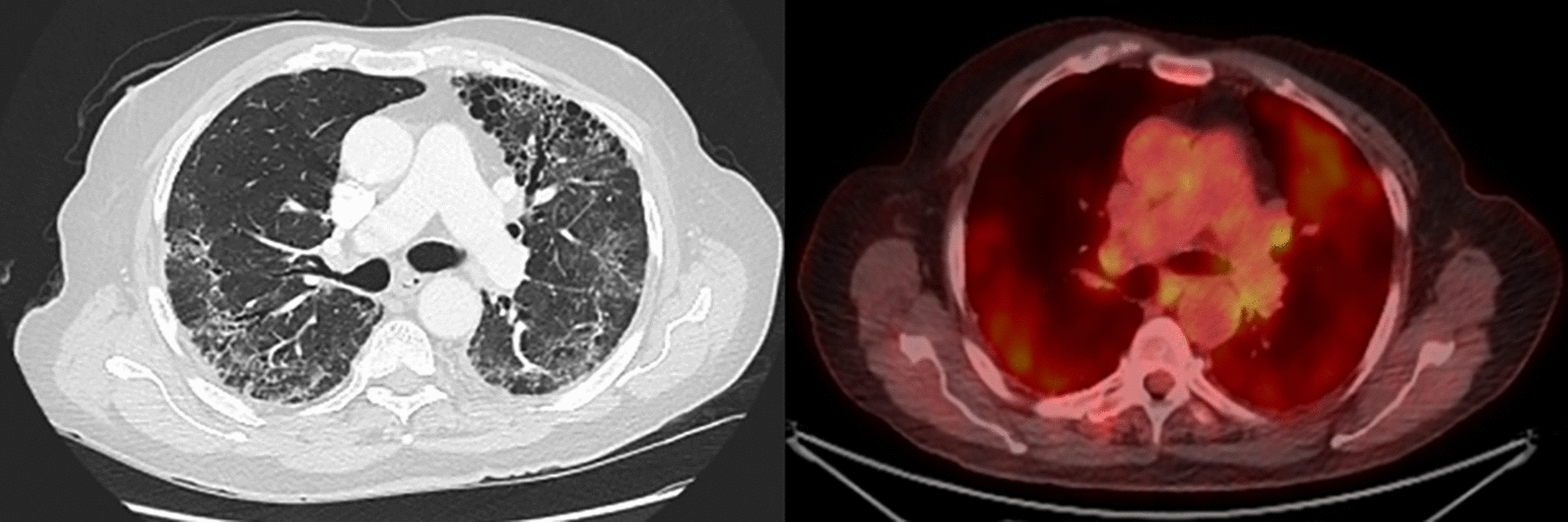
72-year-old man with pulmonary fibrosis and dyspnea on exertion. Axial CT image and fused axial FDG PET/CT demonstrating areas of bilateral heterogeneous FDG uptake, corresponding to honeycombing and reticular opacities

Akaike et al. examined whether ^18^F-FDG PET/CT performed before chemotherapy could predict the onset of an AE of PF in patients with lung cancer and PF treated with chemotherapy. The authors developed a prediction model for AE using logistic regression analyses for the SUV_max_, with univariable analyses showed that the SUV_max_ of contralateral interstitial lesions might be of potential use for predicting the onset of AE in patients with lung cancer and PF in the post treatment setting [71]. The evaluation of two patients with dyspnea using FDG PET/CT has been shown in Figs. 2, 3.

## Ultrasound with second generation harmonic imaging

Medical ultrasound (US) has many advantages over other types of imaging, including that it is inexpensive and quick to perform; it can often be done at the bedside with portable machines, with the newest US probes able to connect directly to smartphones. Further, US does not use ionizing radiation and is noninvasive. US also has the advantage that it can be used for therapeutic purposes, delivering energy to break down nephroliths and thrombosis, accelerate drug absorption through skin, and even ablate tumors [72].

Utilizing different modes of ultrasound and different frequencies can provide a plethora of information and imaging to contribute to patients diagnosis and treatment planning [73]. Second Generation Harmonic Imaging (SGHI) is an offshoot of ultrasound imaging which allows for improved visualization and resolution of microscopic structures without the need for agents used in fluorescence microscopy [74]. This technique has been used to study the tumor microenvironment of breast, ovarian, and skin cancers [74]. By better visualizing and understanding the protein ultrastructure surrounding cancers, biopsy and delivery of chemo- or immunotherapy agents can be optimized.

### Applications for *cancer* imaging

Thoracic US can provide a quick and low-cost means of examining pulmonary structures, though it is not as widely used as abdominal US because normally aerated lung parenchyma is not visualized well on ultrasound. While US might not surpass CT as the gold standard for detection of lung cancer, US can still be a useful supplement.

US can reliably visualize the pleurae, lymph nodes, diaphragm, and anterosuperior mediastinum, making it superior to CT for detecting tumor invasion of the pleurae and thoracic wall, including metastasis to ribs and chest wall, and assessing adjacent lymphadenopathy [75, 76]. US guidance is frequently used for percutaneous biopsy of peripheral lung lesions, with equal reliability and less risk of post-procedural pneumothorax than CT [77].

To enhance the ability of ultrasound to visualize aberrant tissue architecture, polarimetric-SGHI has been used to analyze collagen ultrastructure in multiple malignancies, including lung cancers. Analyses of non-small cell lung cancer under SHG have produced an attenuated SHG signal, indicating disorganization of collagen ultrastructure and potential tumor extracellular matrix; hence, large-area scans of the lung can be conducted to allow for detection and determination of tumors in SHG microscopy [78, 79]. Detection of higher deposition and lower organization of collagen by SGHI has been associated with advanced tumor progression and metastasis [80]. Castor et al. conducted a study using SHG microscopy to compare the collagen fibers in normal tissue, vulvar intraepithelial neoplasia, and vulvar squamous cell carcinoma. The study revealed that organization, uniformity and quantity, of collagen fibers were reduced in both preneoplastic lesions and squamous carcinoma as compared to normal tissue, Though, the difference between preneoplastic lesions and squamous carcinoma was not notable. On the other hand, the presence of distant metastasis correlated with increased collagen uniformity and quantity compared to VSCC without metastasis [81]. SH microscopy can identify three tumor-associated collagen signatures (TACS) at different stages of tumor progression. TACS-1 represents dense collagen with no particular arrangement surrounding early-stage tumors. As the tumor progresses, TACS-2 appears, with collagen fibers wrapping around tumors parallel to the tumor-stromal barrier. TACS-3 emerges in later stages, with fibers placed perpendicularly to the stromal barrier and aligning with cell invasion direction [82].

### Applications for fibrosis imaging

Ultrasound is a useful tool for diagnosing IPF, owing to its capacity for visualizing pleural effusion or lung consolidation [83]. US physics also allow for the detection of altered parenchyma density from loss alveolar air or increased interstitial fluids [83]. However, Yan et al. found in 2021 that US is not as reliable a screening tool for lung fibrosis as CT; they found that lung US had 93% sensitivity and 73% specificity, whereas chest CT had 100% sensitivity and 82% specificity [84]. Meta-analyses suggest that lung US may be more sensitive than radiographs for detecting pleural effusion, pneumonia, and pneumothorax [85]. Thus, US could serve as a potential screening tool in patients with suspected interstitial lung disease or its complications, owing to its non-invasive and properties and lack of ionizing radiation.

Because lung fibrosis occurs in part because of significant changes to the ECM collagen, SGHI is poised to offer unique insight by molecular structural changes in the matrix. It can image collagens I, III, and V, the key molecular players in usual interstitial pneumonia (UIP) and cryptogenic organizing pneumonia (COP), and analyze their macroscopic properties (fiber density and arrangement), and microscopic properties (diameter and density of collagen fibrils) [86, 87]. There are discrete differences between collagen arrangement in UIP and COP. SGHI has found both have elevated collagen I protein, with collagen I being significantly higher in UIP and collagen III more prominent in COP [88]. Future studies of the alterations to the collagen ultrastructure may provide better insight into differences of prognosis and therapy in lung fibrotic diseases.

In another study, Matsuzaki et al. performed a study to investigate fibrosis caused by alcoholic liver injury using SHG microscopy in human postmortem tissue. The study revealed that SHG microscopy effectively identified liver fibrosis and demonstrated a significant association between the SHG images and the fibrosis stage as determined by Sirius Red staining [89].

### Cross-application of US and SGHI between *cancer* and fibrosis

There is substantial value for applying the principles of US and SGHI of lung cancer to lung fibrosis, and vice-versa. Persistent pulmonary fibrosis increases the risk of developing lung cancer, especially squamous cell carcinoma; treating lung cancer in the setting of lung fibrosis also raises the risk of exacerbating the fibrosis [90]. Additionally, lung cancer stage is affected by the degree of fibrosis progression, likely due to them sharing signaling pathways and cellular microenvironments [91].

US provides good visualization of the chest wall and pleurae and can be convenient for monitoring the potential carcinogenesis in patients with lung fibrosis. Should cancerous lesions develop in fibrosis patients, US can also be used to guide management of thoracic malignancy symptoms such as pleural effusion and thoracentesis. Similarly, SHG microscopy can be used to scan regions of abnormal, fibrotic lung tissue for collagen alterations that may signal the genesis of primary lung cancer, or the seeding of metastases permitted by lung fibrosis [92]. Table 2 shows further advantages and limitations of US and SGHI.

**Table 2:** Advantages and limitations of US and SGHI

	Ultrasound	Second generation harmonic imaging
Advantages	Noninvasive	Can be easily integrated with other microscopies (fluorescence, H/E staining)
	Nonionizing radiation	Does not require contrast agents for in vivo imaging
	Rapid and cost-effective	Excellent resolution of protein ultrastructures
Limitations	Limited visualization of deep lung parenchyma	Microscopy is currently limited to collagen, myosin, and tubulin
	Requires skilled technician	Limited field of view and penetration into thicker tissue samples
	Decreased sensitivity versus CT [93]	[94–96]

The TME is recognized as an influential, yet poorly-understood factor in cancer formation and development; it influences angiogenesis, metastasis, and the degree of penetration of therapeutic agents used to treat tumors. The principles of SGHI can be used to better characterize the ultrastructure of the TME, including the arrangement and density of collagen fibers, and how they differ from both fibrotic and healthy parenchyma. Further studies might characterize the TME’s role in the transformation of fibrotic tissue to malignancies, in organs and cancers beyond the lungs, including the liver, kidneys, and bone marrow. Technology is also advancing at a rapid pace; while SGHI instrumentation is commercially available in microscope kits, progress is being made on micro-endoscopic devices incorporating SGHI in the hopes of applying it to laparoscopy and colonoscopy [97].

## MRI imaging (Elastography)

In recent decades, MRI has become increasingly important in cancer diagnosis due to its superior soft tissue contrast and its ability to provide multi-directional, multi-angle, and multi-parameter imaging. The development of fast-sequence MRI has made it possible to obtain high-resolution images for lesion localization and qualitative diagnosis without ionizing radiation. This versatile imaging modality also plays a crucial role in diagnosing and monitoring the progression of fibrosis in multiple organs. Techniques developed for diagnosing fibrosis within organs can also be used to identify tumors.

### Applications for fibrosis imaging

Magnetic Resonance (MR) strain imaging is a technique in echocardiography for measuring the deformation of the heart muscle during the cardiac cycle [98]. It helps identify weakened areas and diastolic dysfunction caused by myocardial ischemia or infarction. It also detects regional heterogeneity in systolic function, as can be seen in bundle branch block [99, 100]. By using strain rate imaging, simultaneous function of different heart regions can be measured and displayed which can indicate the presence and severity of fibrosis.

Cardiac late gadolinium contrast-enhanced magnetic resonance imaging (cMRI LGE), also known as Cardiac LGE CMR, is a specialized method of imaging the heart that employs a gadolinium-based contrast agent during MRI to identify areas of abnormal myocardium which retain contrast to a greater degree than normal tissue [101]. For assessing and measuring myocardial replacement fibrosis and scar tissue, late gadolinium contrast-enhanced CMR stands as the widely accepted and unrivaled benchmark [102].

The utilization of collagen-specific contrast agents in molecular magnetic resonance imaging (mMRI) represents an innovative experimental approach for evaluating myocardial fibrosis. These recently developed contrast agents have demonstrated enhanced visualization capabilities for scar identification and detection of perfusion defects in animal models of myocardial infarction [103, 104]. One of these promising contrast agents is EP-3600, which is a hybrid compound consisting of a small peptide and gadolinium. EP-3600 exhibits the ability to selectively bind to the myocardium, facilitating prolonged, high-contrast, and high-spatial-resolution visualization of perfusion defects in the myocardium [104]. EP-3600 diffuses rapidly into the healthy myocardium and produces a bright MRI signal. In areas with poor blood flow, such as a perfusion defect, diffusion takes longer resulting in a darker MRI signal [104]. EP-3600 achieves reversible binding to myocardial collagen, enabling the differentiation of stress-induced variations in perfusion. This differentiation is accomplished through the myocardium's distinct signal enhancement patterns on subsequent MRI scans, attributable to the differential T1 shortening effect induced by EP-3600 [105].

Perfusion MRI also known as perfusion-weighted imaging, involves utilizing T2- or T2*-weighted MR images sequence to conduct perfusion scans [106]. The resulting data is subsequently processed to generate perfusion maps that provide information about various parameters, including blood volume (BV), blood flow (BF), mean transit time (MTT), and time to peak (TTP). These maps offer valuable insights into the perfusion characteristics of tissues and help in assessing blood flow patterns and potential abnormalities, as can occur with a tumor classification and fibrotic tissue [107, 108].

Ultrashort echo time (UTE)-MRI is used to image tissues with very short T2 relaxation times, such as lungs, to enhance tissue signals [109]. This is valuable tool for detecting small inflammatory and fibrotic lesions in the lungs, which are often missed by conventional proton MRI due to signal loss caused by magnetic susceptibility gradients at the air-tissue interface [110]. UTE-MRI offers the advantage of shorter acquisition times compared to conventional proton MRI [111, 112]. UTE-MRI can also be used for tumor diagnosis due to the specific cellular components that cause a reduction in their T2 relaxation time [113].

Respiratory-gated MRI and self-gated MRI are specialized techniques employed to address the challenges posed by respiratory motion during image acquisition. Both methods are aimed at enhancing image quality and minimizing motion artifacts in areas of the body affected by respiratory motion, such as the lungs and abdomen [114]. Respiratory-gated MRI involves synchronizing the timing of image capture with the patient’s respiratory cycle. By controlling breathing during the recovery period after data acquisition, it effectively reduces respiratory motion artifacts [115]. Self-gated MRI, also known as motion-corrected or motion-resolved MRI, does not rely on external monitoring systems to track respiratory motion. Instead, it corrects motion artifacts based on variations in MRI signal intensity [116]. Both respiratory-gated and self-gated MRI techniques have demonstrated their efficacy in providing more accurate visualization and quantification of lung fibrosis progression in mice treated with bleomycin [117]. AcidoCEST MRI, is a technique that uses a contrast agent to visualize pH changes in tissue, has been adapted for respiratory-gated imaging to measure extracellular pH in lung lesions of IPF [118].

Magnetic resonance elastography (MRE) is an innovative and promising MR imaging technique that offers a noninvasive means of quantifying tissue stiffness in various organs, including the liver. It achieves this by analyzing the propagation of mechanical waves through the tissue [119, 120]. MRE plays a pivotal role in identifying the progressive stiffening of the liver and pancreas that can be attributed to inflammation, fibrosis, and cancer [121]. Wang et al. investigated pancreatic parenchymal stiffness on MRE and found a positive correlation between the severity of chronic pancreatitis and an increase in stiffness. The results indicated that the pancreas stiffness in healthy people had an average of − 1.21 kPa. Compared to healthy individuals, mean stiffness values were higher in patients with mild and moderate/severe pancreatitis with 1.5 and 1.9 kPa respectively [122]. In another study, Higuchi et al. found a direct correlation between HCC risk and liver stiffness increase measured by MRE in 2373 individuals with chronic liver disease [123].

The most promising functional MRI approaches for assessing kidney fibrosis are diffusion weighted (DW)-MRI and blood oxygen level-dependent (BOLD)-MRI [124]. These methods eliminate the need for gadolinium-based contrast agents, which have been linked to the development of nephrogenic systemic fibrosis. BOLD-MRI is used to assess hypoxia, an important factor in renal fibrosis and chronic kidney disease (CKD) progression. In a CKD patient with glomerulonephritis, T2*-based BOLD-MRI revealed significant reductions in oxygenated hemoglobin levels in the renal cortex and medulla, which correlated with the estimated glomerular filtration rate, a measure of overall kidney function [17]. Also, in a rabbit model of unilateral ureteral obstruction (UUO), Woo et al. observed a strong correlation between T2* and the degree of renal fibrosis [125].

### Application of MRI in *cancer*

Spin echo MRI, is a pulse sequence extensively used in MRI to generate high-quality images with excellent tissue contrast. It represents one of the earliest and most widely employed MRI techniques. When comparing lung fibrosis with lung cancer, studies have revealed that spin echo MRI, particularly T1-weighted spin echo MRI, exhibits higher apparent diffusion coefficient (ADC) values and more hypointense appearances in patients with progressive massive fibrosis compared to those with lung cancer [126].

Diffusion weighted imaging (DWI) and ADC values derived from ADC maps have proven effective in diagnosing lung cancers, as cancerous lesions typically impede diffusion due to hypercellularity [127]. Also, these values play a crucial role in distinguishing between benign and malignant lung lesions, correlating with the cancer grade, and monitoring tumor progression [128, 129].

Dias et al. investigated the diagnostic performance of DW-MRI compared to ^18^F-FDG PET/CT on 4463 lesions for differentiation of malignant and benign pulmonary lesions. DW-MRI had better pooled sensitivity and specificity with 83%, 91% compared to ^18^F-FDG PET/CT with sensitivity and specificity of 78% and 81% respectively [130]. In another study reported by Ogihara et al. lung cancer exhibited a higher signal intensity compared to progressive massive fibrosis lesions, particularly on T2-weighted imaging [131].

Another technique to improve diagnosis of liver lesions is the use of gadolinium-ethoxybenzyl-diethylenetriamine-pentaacetic acid (Gd-EOB-DTPA) is a specific MRI contrast agent of liver and bile that is formed by adding fat-soluble ethoxybenzyl (EOB) [132]. Gd-EOB-DTPA shortens T1 relaxation time in normal liver cells causing hyperintensity on T1WI; on the other hand, HCC cells result in relative hypointensity [133]. It can evaluate blood supply and liver cell function of HCC lesions through dynamic enhanced scanning and signal changes in the hepatobiliary phase that can be helpful in HCC diagnosis. Evaluating 570 patients in 10 studies, Wu et al. showed that Gd-EOB-DTPA-enhanced MRI had a sensitivity of 0.95 and specificity of 0.89 for detecting liver cancer under 2.0 cm in patients with chronic liver disease [134].

### Cross-application of MRI techniques between *cancer* and fibrosis

Techniques developed to visualize cancer can be applied to visualize fibrosis, and vice versa. Shin et al. utilized DW-MRI to differentiate between locally recurrent tumors and postsurgical fibrosis after pancreatic ductal adenocarcinoma resection. In this study, DW-MRI was used to evaluate 66 patients who had pancreatic ductal adenocarcinoma resection and postoperative CT showing a soft-tissue lesion. They found higher accuracy and sensitivity for diagnosing locally recurrent tumors in DW-MRI compared to conventional MRI differentiation [135]. In another study, Wang et al. compared DW-MRI with Dynamic Contrast Enhanced MRI to differentiate recurrence or tumor residue from postoperative fibrosis in 11 bladder carcinoma patients. The study found that DW-MRI demonstrated higher reliable diagnostic efficiency, with 100% sensitivity, 81.8% specificity, 92.6% accuracy, and a positive predictive value (PPV) of 88.9% [136].

## Radiomics

Radiomics has been proven to be helpful in early detection and screening of different cancers [137], including lung cancer [138], pancreatic cancer [139], liver cancer [140], breast cancer [141], and other organ cancers. For common cancers such as breast cancer, radiomics can be valuable in risk prediction, even in screening imaging [142]. After diagnosis, radiomics can be helpful in the assessment of tumor grade [143, 144], nodal involvement [145, 146], and distant metastasis [147]. Cancer survival outcomes and treatment responses are also predictable using radiomics [148–150]. Radiomics can also predict metastasis or cancer recurrence by identification of specific textural features of cancer stem cells, which are thought to play a critical role in cancer recurrence and metastasis [151].

### Applications for *cancer* imaging

Radiomics can be used at various stages of the screening and diagnosis process. Torres et al. developed a radiomic method for lung cancer screening. They studied chest CT of 60 patients with a single pulmonary nodule (SPN) sized 8–30 mm and a non-small cell lung carcinoma diagnosis. To identify features correlated with malignancy, they defined a region of interest (ROI) and Otsu threshold using the segmentation method, extracted radiomics features such as shape and textural features, and used PyRadiomics to identify the features significantly correlated to malignancy evaluated by T-test. They then entered chosen features as an input of feedforward neural network. Sensitivity, specificity, and slice diagnostic index were evaluated for each model, and the model with the highest sensitivity, specificity, and slice diagnostic index were chosen. For an independent set of patients, the best model had 100% sensitivity, 83% specificity, and area under the curve (AUC) of 0.94 for malignancy detection [152].

La Forgia et al. developed a radiomic approach to predict the histological outcome of breast cancer using contrast-enhanced breast cancer mammography (CESM). They used the CESM image features and molecular parameters of 68 breast lesions extracted from 52 patients. Ultrasound-guided biopsy sampling was performed for lesions to assess the expression of estrogen receptor (ER), progesterone receptor (PR), and human epidermal growth factor receptor 2 (HER2). They segmented the tumors, extracted radiomic features, and evaluated the statistical correlation between each radiomic feature and histological outcome (e.g., ER, PR, HER2). This study revealed that breast tumor histological outcome and molecular subtypes can be differentiated using features extracted from CESM [153].

Given that chronic fibrosis might lead to cancer, especially in the lung and liver [3, 154], identifying fibrotic texture can indicate a precancerous lesion. Fibrotic tissue might be detected as a texture with increased heterogeneity and reduced correlation with neighboring pixels [155, 156]. These features can be recognized by radiomics developed for texture analysis [157, 158]. In addition to detection, radiomics is practical in fibrosis staging [159], monitoring of disease progression, and prognosis prediction [160].

### Applications for fibrosis imaging

Studies have evaluated the performance of radiomic assessment of fibrosis. Park et al. developed and verified a radiomic-based method for fibrosis staging using MRI. This study included 436 patients with pathology-proven liver fibrosis referred for gadoxetic acid-enhanced hepatobiliary phase imaging. Serum fibrosis tests were also done for patients to assess the aspartate transaminase–to-platelet ratio index (APRI) and the fibrosis-4 index. These non-invasive diagnostic tests are potential alternatives in liver biopsy for diagnosing and managing liver fibrosis and cirrhosis. First, the liver and spleen were manually segmented by ROI drawing. Then, histogram and textural features were extracted from ROIs, and finally significantly relevant features were chosen for modeling using logistic regression with elastic net regularization. The final model in this study was a binary classification model for differentiating F0–F2 and F3–F4. The diagnostic performance of the model for fibrosis staging in clinically significant fibrosis, advanced fibrosis, and cirrhosis was assessed, revealing that its sensitivity was between 80.3 and 87, specificity was 73.8–84.5, and accuracy was 80.9–82.1 [161].

Refaee et al. developed a radiomic-based, DL approach to differentiate IPF from non-IPF disease using HRCT of 474 patients. A team of specialists confirmed each patient's diagnosis, and a biopsy was performed for ILD inconsistent with IPF. An automated whole lung segmentation was performed followed by radiomic feature extraction, then significantly relevant features were used for radiomic modeling using a random forest classifier. DL was also performed. The accuracy of the radiomic model (76.2 ± 6.8%), DL model (77.9 ± 4.6%), and ensemble radiomic-DL model (85.2 ± 2.7%) were assessed [162].

### Cross-application of radiomics between *cancer* and fibrosis

Fibrosis is a hallmark of cancer [1, 154]; both affect tissue architecture, and they differ in the tissue texture is affected. Therefore, the assessment of textural radiomic features in both can be helpful in diagnosis of each and differentiation between the two. Radiomic machine learning (ML) algorithms for fibrosis texture analysis can also be applied for cancer analysis, highlighting the potential for cross-disease application of radiomics techniques. Liang et al. aimed to evaluate the ability of lung CT texture analysis to predict lung cancer risk stratification in a cohort of 116 IPF patients. The study included a training cohort of 92 patients with both cancerous and non-cancerous conditions and a validation cohort of 24 patients with the same conditions. The selected radiomics features were energy and kurtosis, which respectively measure the intensity of voxel values in the image and the degree of cellularity within a tumor. The authors also performed risk factors such as gender, age, smoking, and emphysema. They found that radiomics features based on texture can distinguish between IPF patients who have developed cancer and those who have not. Combining radiomics features with risk factors can improve diagnostic accuracy [163].

## Artificial intelligence

**Fig. 4:**
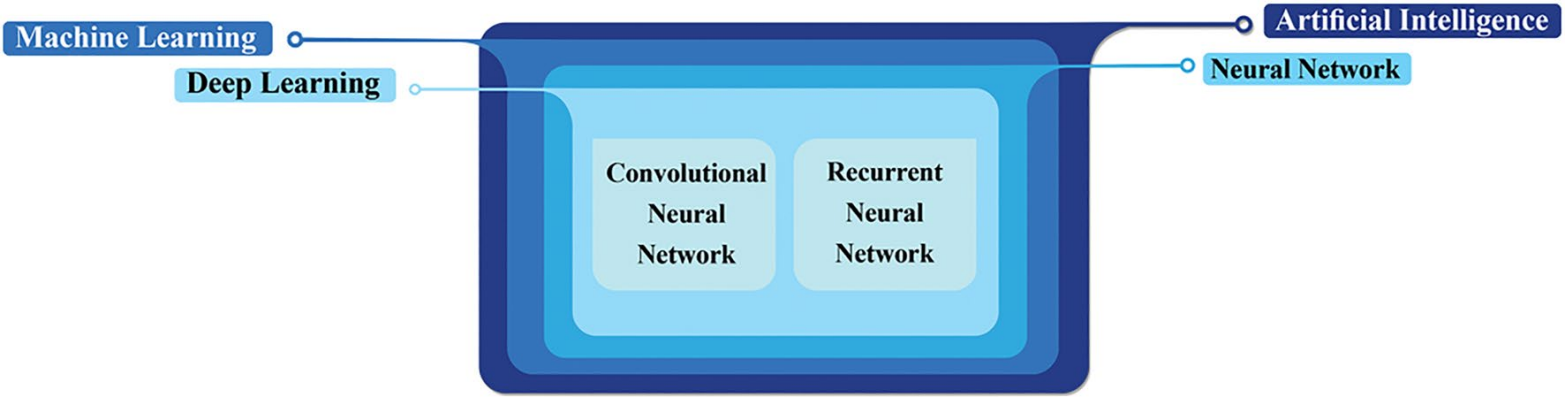
Relationship between artificial intelligence, machine learning, and deep learning

AI technology is an example of sophisticated computational science that utilizes advanced analytical and predictive capabilities to manage challenges across various fields of medicine and has been applied to both the study of fibrosis and cancer research [164]. ML and DL are two fields within the larger scope of AI. ML is known as a subfield of AI that utilizes datasets to acquire knowledge on how to execute a specified task, so as to construct data models and algorithms that can be applied to subsequent cases, used in many tasks such as analysis, classification, prediction, etc. [165–167]. DL is a subtype of ML that leverages neural networks to process vast amounts of data and make intricate decisions [168, 169]. Neural networks resemble the nervous system, with interconnected neuron nodes forming the network that consists of input, hidden, and output layers [170]. As the data flows through this network, they are processed through the layers of neurons by using mathematical operations that enable the network to learn [171]. DL has multiple layers that take training data as input and perform various tasks such as feature extraction and classification [172, 173]. Convolutional-neural-networks (CNNs) and recurrent-neural-networks (RNNs) are two popular DL architectures widely used for analyzing different types of data. CNNs include three layers: (1) convolutional for extraction of feature map from an image, (2) pooling for performing filters on features map and down-sampling to reduce the size of this, and (3) fully connected layers for classification or regression, thus frequently used for image analysis and classification; while RNNs due to their internal memory can remember their previous input and use this memory to process a sequence of inputs, so often used for text analysis, process time series, and sequential data for prediction of an outcome [174–176]. The relationship is shown in Fig. 4.

Recently, attention has been paid to the potential benefits of AI in different fields, including prediction, early diagnosis, tumor staging, prognosis, treatment, and more [177–179] Table 3. Radiologists can utilize AI as a complementary tool to aid their work. Here, we will explore how AI-based models can assist in detecting and managing cancer and fibrosis, both individually and as interrelated pathologies.

**Table 3:** Summary of the use of AI in the fields of cancer and fibrosis

	Interest	Images	Models	Results
Yoo et al. [182]	Pulmonary nodules	Chest radiographs	DCNN based on ResNet-34	For nodule diagnosis, the AI had better sensitivity 96% and specificity 93.2% compared to radiologists
Khan et al. [185]	Pulmonary nodules	CT images	AdaBoost-SNMV-CNN with LIDC-IDRI and ELCAP	Lung nodules detection on LIDC-IDRI had 93% sensitivity, and 92% specificity; on ELCAP, it achieved 100% sensitivity with 98% specificity
Yang et al. [186]	Focal liver lesions	B-mode ultrasonography	DCNN-US based on ResNet-CNN	Had higher sensitivity and specificity in identifying FLLs compared to radiologists in identifying FLLs
H-T Hu et al. [187]	Focal liver lesions	Contrast-enhanced ultrasound images	CEUS based ResNet	Showed 91% accuracy compared to radiologists for differentiation between benign and malignant FLLs
Cao SE et al. [188]	Liver lesions	DCE-CT images	MP-CDN	Showed an acceptable performance with mean accuracy of 81.3% in classifying various types of FLLs
Hamm et al. [189]	Liver lesions	MR images	CNN model	Demonstrated 92% sensitivity, and 98% specificity compared with radiologists
Nishikiori et al. [191]	Chronic fibrosing in ILDs	Chest radiograph images	DCNN and using DenseNet121 architecture	Showed 0.979 AUC to identify chronic-fibrosing ILDs
Furukawa et al. [192]	IPF	HRCT images	combination of deep fully-convolutional-neural-network FCN-Alexnet and SVM	Had accuracy of 83.6% to identify IPF from ILDs
Pawar and Talbar [193]	Classifying into six ILD classes	HRCT images	Combination of c-GAN, ResNet50 and SVM	Had accuracy of 89.39% to classifying ILD
Xie et al. [195]	Liver fibrosis	US images	CNN models	GoogLeNet had the better performance with an accuracy of 95.29% to identify fibrosis on liver

### Applications for *cancer* imaging

One of the most exciting opportunities to employ AI to improve healthcare is for improved detection and diagnosis of precancerous and early cancerous lesions. For example, early detection of pulmonary nodules is necessary to reduce lung cancer-associated mortality [180, 181]. Multiple groups have employed AI to enhance pulmonary nodule detection. For example, Yoo et al. utilized an AI algorithm called Lunit INSIGHT CXR to analyze chest radiographs. The algorithm used a deep convolutional neural network (DCNN) based on the ResNet-34 architecture. The training data for the algorithm included both digital and computed radiographs of 12,408 abnormal images that were read by experienced radiologists and 72,704 normal images that were collected from multiple centers in South Korea. A subset of 577 participants out of 5485 were chosen for the nodule dataset. Within one year, 48 out of 5485 participants were diagnosed with cancer. Of those, 34 had visible malignant nodules and 14 had no visible lesions. In addition, 3 of the 48 diagnosed with cancer had other manifestations of lung cancer on their chest images. The results showed that the AI algorithm outperformed radiologists for the detection of noncalcified and malignant pulmonary nodules on digital radiographs. For nodule diagnosis, the AI in the nodule data set exhibited better sensitivity (96% vs 88%) and specificity (93.2% vs 82.8%) compared to radiologists. On the other hand, for malignant diagnosis in the full data set, the AI had better performance with 100% sensitivity vs 94.1% for radiologists, while the specificity was 90.9% vs 91% respectively [182]. Chen developed an improved 3D U-Net model by combining CNN, RNN, and long short-term memory (LSTM), an improved RNN variant with memory blocks that better preserve long-range dependencies and enhances previous data recall for CT images to diagnose lung nodules [183]. The detection rate of this model was 100% compared with radiologists with detection of 99.99%. Compared to improved 3D U-net model output to two-person readings of H&E-stained slices from 652 patients' lung lesions, the system achieved an accuracy rate of 92.3% for predicting malignant lung nodules and 82.8% for benign lung nodules [184]. Khan et al. used an Adaptive Boosting Self-Normalized Multiview Convolution Neural Network (AdaBoost-SNMV-CNN) to detect lung cancer nodules in CT scans. It has been trained and tested with LIDC-IDRI (Lung Image Database Consortium and Image Database Resource Initiative) and ELCAP (Early Lung Cancer Action Program) datasets. On LIDC-IDRI dataset, this model was able to detect lung nodules with 92% accuracy, 93% sensitivity, and 92% specificity; on ELCAP, it achieved 99% accuracy and 100% sensitivity with 98% specificity compared to other models that were used in similar previous study [185].

Similarly, AI has been applied to the field of liver cancer to assist radiologists in diagnosis. Yang et al. developed DCNN of US (DCNN-US) models based on ResNet-CNN architecture that were trained using manually segmented planar regions of interest from images of liver background or lesion. These models have been used in B-mode ultrasonography to identify malignant and benign focal liver lesions (FLLs). This study suggested that DCNN-US has higher sensitivity and specificity in identifying FLLs compared to skilled radiologists and is comparable to detection rates with contrast-enhanced CT [186]. In another study, H-T Hu et al. used contrast-enhanced ultrasound (CEUS)-based ResNet architecture for differentiation between benign and malignant FLLs. This model shows 91% accuracy compared to radiologists with 82% to 86.7% accuracy. It can help radiologists to improve their performance [187]. An automated multiphase-convolutional-dense-network (MP-CDN) developed by Cao SE et al. was utilized to classify liver lesion images obtained from multiphase dynamic contrast-enhanced CT (DCE-CT). Training and testing included 410 (105 abscesses, 128 benign non-inflammatory FLLs, 89 metastases, 88 hepatocellular carcinomas (HCCs) and 107 FLLs (and 27 abscesses, 34 benign non-inflammatory FLLs, 23 metastases and 23 HCCs), respectively. The model had an acceptable performance with mean accuracy of 81.3% in classifying various types of FLLs, including abscesses, hemangiomas, focal nodular hyperplasia, adenomas, HCC, and metastases. The accuracy, specificity, and sensitivity of differentiating each category were as follows: abscesses (0.925, 0.963, 0.815), benign non-inflammatory FLLs (0.86, 0.918, 0.735), metastases (0.925, 0.905, 1.0), and HCC (0.916, 0.964, 0.739) [188].

Similarly, a CNN model was developed by Hamm et al. for the classification of six types of liver lesions using imaging characteristics from multiphasic MRI. This study was trained with 434 and tested with 60 liver lesions of MR images. The types of lesions included cyst, hemangioma, focal nodular hyperplasia, intrahepatic cholangiocarcinoma, HCC, and colorectal metastasis. This study showed an accuracy of 92%, sensitivity of 92%, and specificity of 98% of this model compared with radiologists’ reads, which had an average sensitivity of 82.5% and specificity of 96.5% [189].

### Applications for fibrosis imaging

Similar methods can be applied to study fibrosis of organs and improve outcomes for patients. Classically, PF is followed radiographically. Given the importance of progressive disease in patients with PF, assessment by AI may give a more accurate quantification of change over time and therefore patients’ progress than visual assessment [190]. Nishikiori et al. utilized an algorithm developed by DCNN and using DenseNet121 architecture to identify chronic-fibrosing ILDs in chest radiograph images. The algorithm demonstrated a 0.979 AUC and was comparable with radiologists’ and pulmonologists’ reads [191]. Furukawa et al. used a combination of deep fully-convolutional-neural-network FCN-Alexnet and support vector machine (SVM) to construct their algorithm to identify IPF from ILDs on HRCT images. The result was an accuracy of 83.6% [192]. In a similar study, Pawar and Talbar developed an algorithm that used the combination of conditional generative adversarial network (c-GAN) for segmentation of HRCT images to remove the unnecessary background of images and ResNet50 architecture and SVM for classifying result images into six ILD classes, including normal, fibrosis, micronodules, emphysema, ground glass, and consolidation. This algorithm yielded an accuracy rate of 89.39%, better than the previous study [193].

If liver fibrosis diagnosis occurs in the early stage, it can be reversible with treatment, so it is crucial to diagnose fibrosis in real-time [194]. Compared to other imaging methods, ultrasonography is more available and cost-effective for screening and follow-up of liver fibrosis, and it is safe given the lack of ionizing radiation. Xie et al. used GoogLeNet, AlexNet, GG-16, and VGG-19 architectures to develop a CNN model to identify fibrosis on liver US images. Out of these four network models, GoogLeNet had the best performance with an accuracy of 95.29% compared to the other architectures to classify and analyze liver US images, and the accuracy of the remaining three models was 38.95%, 67.28%, and 86.76%, respectively [195].

### Cross-application of artificial intelligence between *cancer* and fibrosis

As mentioned before, AI applications in medicine are advancing rapidly. Although AI algorithms, especially ML, need a large number of medical images for training and better performance [172], it has been demonstrated that they apply as a supplement tool in the field of both fibrosis and cancer.

For instance, an AI technology initially developed for assessing fibrosis has been effectively used to diagnose and track cancer. Gómez-Zuleta et al. used liver fibrosis index (LFI) measured during endoscopic ultrasonography, which was improved by AI and validated for liver fibrosis diagnosis, to evaluate whether the LFI can differentiate three types of pancreatic tissues: endoscopically normal, fatty pancreas, and patients diagnosed with pancreatic cancer confirmed by cytology. They found that the LFI was effective in differentiating these three types of pancreatic tissues non-invasively [196]. Vuppalanchi et al. evaluated 152 patients with primary sclerosing cholangitis (PSC), which may lead to cholangiocarcinoma and liver failure, for an average of three years. As a retrospective study,102 patients were enrolled in the training cohort with an additional 50 in the validation group, and finally, 34 patients experienced liver transplantation and death. In this study, they used three predictive criteria including MRCP + , which is a new post-processing technique based on AI, that allows quantification of magnetic resonance cholangiopancreatography (MRCP) data, and two serum markers including total bilirubin and aspartate aminotransferase. They generated MRCP + metrics, that is ratio of the bile ducts have a diameter between 3 and 5, with total bilirubin and aspartate aminotransferase (M + BA) composite risk score to predict survival in PSC patients. Patients with a high probability of liver transplantation and death were identified with the area under the receiver operator curve (AUROC) of 0.86 by M + BA compared to the current blood-based risk score (Mayo risk score) [197]. However, more research is needed to identify if these patients would also be at a higher risk of malignancy based on AI models.

## Conclusion

The imaging modalities described above comprise a comprehensive set of tools for clinicians and researchers to be able to diagnose and monitor the progression of diseases that result from enhanced fibrogenesis, including cancer. While clinically these imaging modalities may appear in opposition with each other, given that the ACR appropriateness criteria ranks imaging modalities from most to least appropriate, giving the appearance of “better” and “worse” choices, the truth is that the present problem-solving tools to advance the study of a given disease in different contexts with different strengths and weaknesses. CT can be obtained easily and nearly universally, often providing the first insight into an individual’s disease process. PET can enhance the imaforation obtained by CT by providing functional information based on the tracer administered. Where both of these techniques fail in soft tissue discrimination which may be an important characteristics in the diagnosis of cancer and fibrotic diseases, MRI is excellent in providing this information. Ultrasound is more easily obtained, and while resolution may be decreased compared to other modalities, the benefit of repeated monitoring and the used of advanced ultrasonographic techniques may provide enhanced information based on tissue characteristics, leading clinicians toward one diagnosis versus another. Finally, the application of radiomic and artificial intelligence techniques to each of these has the potential to extract more information from the images obtained and therefore provide better diagnostic clarity. In contrary to being in opposition, these multiple techniques represent a complimentary set of tools to move toward early and accurate diagnosis of diseases characterized by fibrogenesis.

In order to best diagnose diseases with fibrotic pathophysiology, these techniques have been employed and continue to advance the field. Here, we have reviewed them and how techniques originally developed for each can advance the diagnosis of the other. These advanced methods can improve the screening process, enable early diagnosis, increase accuracy and rate of diagnosis, have better staging performance, predict tumor progression and metastasis, forecast mortality and survival rates in fibrosis and cancer, may provide a better means for both therapy and treatment-response monitoring, and reduce complications. While fibrotic disease may be most easily recognized in the lungs and liver, there is increasing recognition that other organs such as the breasts and thyroid may have similar pathophysiologic cross-over between cancerous and fibrotic disease states. As research advances in other organ sites, this relationship is becoming increasingly recognized as a major pillar of both diseases. Despite these advances, further studies are needed to confirm the results in broader populations and implement these novel diagnostic techniques more universally. By understanding the commonalities of these conditions and developing novel imaging technologies for each in parallel, we can improve diagnosis and treatment of cancer and fibrosis resulting in better outcomes for patients.

## Data Availability

Not applicable.

## List of abbreviations

PET: Positron emission tomography
MRI: Magnetic resonance imaging
SGHI: Second generation harmonic imaging
US: Ultrasound
AI: Artificial intelligence
CT: Computed tomography
FAPI: Fibroblast activation protein inhibitor
FAP: Fibroblast activation protein
TME: Tumor microenvironment
DL: Deep learning
CAF: Cancer-associated fibroblast
TGF-β: Transforming growth factor β
ECM: Extracellular matrix
HU: Hounsfield unit
PF: Pulmonary fibrosis
^18^F: 18-Fluorine
FDG: Fluorodeoxyglucose
SUV_max_: Maximum standardized uptake value
MTV: Metabolic tumor volume
TLG: Total lesion glycolysis
^18^F-FLT: ^18^F-fluorothymidine
^18^F-FMISO: ^18^F-fluoromisonidazole
[^18^F]FSPG: (S)-4- (3-^18^F-Fluoropropyl)-L-Glutamic Acid
PMSA: Prostate-specific membrane antigen
2-[^76^Br]-BAMP: 2-76Br-bromo-α-methyl-L-phenylalanine
uPAR: Urokinase-type plasminogen activator receptor
ccRCC: Clear cell renal cell carcinoma
HR: High resolution
TBR: Target-to-background ratio
MLV: Metabolic lung volume
ILD: Interstitial lung disease
GAP: Sex-age-physiology
TF: Tissue fraction
qCT: Quantitative CT
^18^F-FBEM: (18) F-4-fluorobenzamido-N-ethylamino-maleimide
CCR2: Chemokine receptor 2
GPVI: Glycoprotein VI
IPF: Idiopathic pulmonary fibrosis
AE: Acute exacerbations
SHG: Second-harmonic generation
UIP: Usual interstitial pneumonia
COP: Cryptogenic organizing pneumonia
mMRI: Molecular magnetic resonance imaging
UTE: Ultrashort echo time
MRE: Magnetic resonance elastography
BOLD: Blood oxygen level-dependent
CKD: Chronic kidney disease
ADC: Apparent diffusion coefficient
DWI: Diffusion weighted imaging
Gd-EOB-DTPA: Gadolinium-ethoxybenzyl-diethylenetriamine-pentaacetic acid
SPN: Single pulmonary nodule
ROI: Region of interest
AUC: Area under the curve
CESM: Contrast-enhanced breast cancer mammography
ER: Estrogen receptor
PR: Progesterone receptor
HER2: Human epidermal growth factor receptor 2
APRI: Aspartate transaminase–to-platelet ratio index
ML: Machine learning
CNN: Convolutional-neural-network
RNN: Recurrent-neural-network
DCNN: Deep convolutional neural network
LSTM: Long short-term memory
DCNN-US: Deep convolutional neural network of ultrasound
FLL: Focal liver lesion
CEUS: Contrast-enhanced ultrasound
MP-CDN: Multiphase-convolutional-dense-network
HCC: Hepatocellular carcinoma
SVM: Support vector machine
LFI: Liver fibrosis index
PSC: Primary sclerosing cholangitis
MRCP: Magnetic resonance cholangiopancreatography
